# Melatonin ameliorates renal fibroblast‐myofibroblast transdifferentiation and renal fibrosis through miR‐21‐5p regulation

**DOI:** 10.1111/jcmm.15221

**Published:** 2020-04-03

**Authors:** Ningning Li, Zhan Wang, Fenglan Gao, Yanfei Lei, Zhenzhen Li

**Affiliations:** ^1^ Department of Pathology Henan Medical College Zhengzhou China; ^2^ Department of Surgery Henan Medical College Zhengzhou China; ^3^ Department of Traditional Chinese Medicine Henan Medical College Zhengzhou China; ^4^ Medical Research Center The First Affiliated Hospital of Zhengzhou University Zhengzhou China

**Keywords:** fibroblast‐myofibroblast transdifferentiation, melatonin, microRNA‐21‐5p, renal fibrosis, transforming growth factor (TGF)‐β1

## Abstract

Fibroblast‐myofibroblast transdifferentiation (FMT) is widely recognized as the major pathological feature of renal fibrosis. Although melatonin has exerted antifibrogenic activity in many diseases, its role in renal FMT remains unclear. In the present study, the aim was to explore the effect of melatonin on renal FMT and the underlying mechanisms. We established the transforming growth factor (TGF)‐β1 stimulated rat renal fibroblast cells (NRK‐49F) model in vitro and unilateral ureteral obstruction (UUO) mice model in vivo. We assessed levels of α‐smooth muscle actin (α‐SMA), col1a1 and fibronectin, STAT3 and AP‐1, as well as miR‐21‐5p and its target genes (Spry1, PTEN, Smurf2 and PDCD4). We found that melatonin reduced the expression of α‐SMA, col1a1 and fibronectin, as well as the formation of α‐SMA filament in TGF‐β1‐treated NRK‐49F cells. Meanwhile, melatonin inhibited STAT3 phosphorylation, down‐regulated miR‐21‐5p expression, and up‐regulated Spry1 and PTEN expression. Moreover, miR‐21‐5p mimics partially antagonized the anti‐fibrotic effect of melatonin. For animal experiments, the results revealed that melatonin remarkably ameliorated UUO‐induced renal fibrosis, attenuated the expression of miR‐21‐5p and pro‐fibrotic proteins and elevated Spry1 and PTEN expression. Nevertheless, agomir of miR‐21‐5p blocked the renoprotective effect of melatonin in UUO mice. These results indicated that melatonin could alleviate TGF‐β1‐induced renal FMT and UUO‐induced renal fibrosis through down‐regulation of miR‐21‐5p. Regulation of miR‐21‐5p/PTEN and/or miR‐21‐5p/Spry1 signal might be involved in the anti‐fibrotic effect of melatonin in the kidneys of UUO mice.

## 1 INTRODUCTION

Globally, chronic kidney disease (CKD) has gradually grown a primary economic burden in many countries and seriously threatens life quality of patients. The prevalence of CKD is predicted to be 8%‐16% all over the world, even higher afterwards.^1^ The present counter measure for remedying CKD mainly includes the control of systemic blood pressure, dyslipidaemia and hyperglycaemia.^2^ Effective therapeutic strategies for CKD are still lacking in the clinical treatment. As a consequence, efforts to find more new therapeutic strategies that treat CKD or retard progression of CKD are urgently needed.

Renal fibrosis is a common pathological process from the progression of all CKD to end‐stage renal disease.^3^ As the main effector cells, myofibroblasts play an important role in the process of renal fibrosis, and its large accumulation in the renal interstitial is the main pathological feature of fibrosis.^4^ During the development of progressive fibrosis, myofibroblasts appear to acquire smooth muscle cell characteristics, mainly expressing alpha‐smooth muscle actin (α‐SMA). Accordingly, α‐SMA‐positive myofibroblasts are closely related to interstitial extracellular matrix aggregation and fibrosis‐induced tissue disruption. Extensive investigations suggest that myofibroblasts may generate from activated resident fibroblasts or pericytes^5^; or perivascular fibroblasts,^6^ or circulating bone marrow‐derived fibrocytes^7^; or epithelial and endothelial cells, which go through epithelial‐to‐mesenchymal transition (EMT)^8^ and endothelial‐to‐mesenchymal transition (EndoMT),^9^ respectively. Recent evidence indicated that resident renal fibroblasts tend to directly differentiate to form myofibroblasts under the pro‐fibrotic stiff microenvironment, for example the stimulation of transforming growth factor (TGF)‐β1.^10^ Regardless of the origin of myofibroblasts, blocking the pathways that promote myofibroblast expansion should be an option worth considering in treating renal fibrosis.

It has been demonstrated that expression of miR‐21 greatly increased during renal fibrogenesis.^11^, ^12^ Overexpression of miR‐21 has been discovered in the injured kidney and closely related to the stage of renal fibrosis.^11^, ^12^, ^13^ Additionally, knock‐down of miR‐21 in renal cells acquires good effects against renal fibrosis.^14^ Even more intriguingly, there is some evidence that a prominent elevation of miR‐21 primarily located in myofibroblasts in the kidneys of unilateral ureteral obstruction (UUO) mice and patients’ kidneys with severe renal fibrosis.^15^ Collectively, these findings imply that miR‐21 plays an important role in renal fibrosis.

Melatonin is an indolamine product produced by the pineal gland and is believed to have a significant effect on regulating a series of biological functions, including anti‐inflammation, anti‐oxidation, inhibition of intrarenal renin‐angiotensin system (RAS) activation and preservation of renal function.^16^, ^17^ Our recent findings have also shown that melatonin administration can potently protect against bilateral ureteral obstruction‐induced renal injury.^18^ However, as far as we know, there is currently no information about melatonin in the context of fibroblast‐myofibroblast transdifferentiation (FMT) during renal fibrosis. In the present study, the TGF‐β1‐treated rat renal fibroblast cells (NRK‐49F) and UUO mice were used to investigate whether melatonin could attenuate renal FMT and whether the miR‐21‐5p signal might serve a role in melatonin‐induced renal protective effect.

## 2 MATERIALS AND METHODS

### 2.1 Main reagents

Melatonin was acquired from Sigma (Cat. No M5250, SIGMA‐ALDRICH). Recombinant human TGF‐β1 was purchased from R&D Systems. Stattic was purchased from Selleckchem (Cat. No S7024, Selleckchem). Control mimics, miR‐21‐5p mimics, negative control siRNAs, Spry1 siRNA and PTEN siRNA were designed and synthesized by Shanghai GenePharma. Control agomir and mmu‐miR‐21‐5p agomir were also purchased from Shanghai GenePharma.

### 2.2 Cell culture and treatment

Rat renal fibroblast cell line NRK‐49F were purchased from the American Type Culture Collection and cultured in Dulbecco's modified Eagle's medium (DMEM) containing 5% foetal bovine serum (FBS) (Gibco), 100 IU/mL penicillin and 100 IU/mL streptomycin at 37°C in a 5% CO_2_ atmosphere. Cells were seeded on 6‐well plate at a density of 2 × 10^5^ cell/well and allowed to adhere 24 hours. Afterwards, the medium was changed to DMEM containing 0.1% FBS. Cells were incubated with TGF‐β1 (2 ng/mL) alone, or TGF‐β1 (2 ng/mL) with different concentrations of melatonin for the indicated periods before harvesting. For the specific experiment, the medium was supplemented with 10 µmol/L Stattic dissolved in dimethyl sulphoxide (DMSO) (SIGMA‐ALDRICH) for the indicated period before collection.

The control mimics, miR‐21 mimics, negative control siRNA for Spry1, Spry1 siRNA, negative control siRNA for PTEN or PTEN siRNA were transfected with Lipofectamine RNAiMAX Reagent (CAT No: 13778150, Thermo Fisher Scientific) based on the manufacturer's protocols before TGF‐β1 administration or co‐treatment with melatonin.

### 2.3 Cell viability assay

Cell viability was examined by CCK‐8 assay (Cat. No.: HY‐K0301, MCE) following the instructions of manufacturer. Briefly, about 8000 cells were seeded into each well of 96‐well cell plate for 24 hours. Then, cells were incubated with different doses of melatonin. After 3 days, 10 µL of CCK‐8 solution was supplemented to each well, and the absorbance was measured at 450 nm in the microplate reader after 4‐ hours incubation. This experiment was repeated independently three times.

### 2.4 Luciferase reporter assay

The pGL3‐AP‐1 firefly luciferase reporter plasmid and renilla luciferase control reporter vector were transfected into NRK‐49F cells with Lipofectamine 2000 reagent following the manufacturer's protocols. Twenty‐four hours after transfection, the cells were serum free in DMEM with 0.1% FBS overnight, then treated with TGF‐β1 (2 ng/mL) alone or TGF‐β1 combined with melatonin (10 µmol/L) for 48 hours and lysed in passive lysis buffer. Bioluminescence signals were determined using dual‐luciferase reporter assay system (Promega). The signal intensity from the firefly luciferase reporter gene was normalized to that of the renilla luciferase reporter vector.

### 2.5 Immunofluorescence staining

NRK‐49F cells were treated with recombination TGF‐β1 (2 ng/mL) alone or TGF‐β1 combined with melatonin (10 µmol/L) for 48 hours. The cells were fixed with ice‐cold 1:1 methanol/acetone solution for immunofluorescence analysis. Afterwards, these sections were blocked with 10% normal goat serum, incubated with 100 nmol/L working stock of rhodamine phalloid at 4°C in the dark overnight. After washing with PBS three times, the slides were counterstained with 200 µL of 100 nmol/L 4,6‐diamidino‐2‐phenylindole (DAPI) in PBS. In the end, images were collected on a Zeiss laser‐scanning confocal microscope (Olympus). F‐actin level in every single experimental group was quantified using Image Pro Plus 6.0 analysis software by taking measurements of the average pixel intensities in 5‐10 random fields.

### 2.6 Establishment of an UUO animal model

Seven‐week‐old male C57BL/6 mice weighing 23‐28 g were purchased from Zhengzhou University Laboratory Animal Center and then were kept in separate cages in a light‐ and temperature‐controlled environment. The animal study in this experiment got consent from the Animal Ethics Committee of the First Affiliated Hospital of Zhengzhou University. Procedures involving animals and their care were completed in accordance with the National Institutes of Health guidelines and were approved by Animal Care and Use Committee of the First Affiliated Hospital of Zhengzhou University.

Unilateral ureteral obstruction (UUO) was conducted using an established procedure as described in previous published papers.^18^ In brief, mice were anaesthetized with isoflurane inhalation, and the left proximal ureter was exposed and ligated with 6‐0 silk sutures. Mice in sham group were subjected to operations that were identical to the ones done for the mice with UUO except that the ureters were not ligated. Forty‐eight weight‐matched C57BL/6 mice were randomly allocated to six groups (n = 8).

1: sham group;

2: UUO 7 day group (UUO);

3: UUO 7 day plus melatonin group (UUO + Mel);

4: UUO 7 day plus melatonin and control agomir group (UUO + Mel +NC);

5: UUO 7 day plus melatonin and mmu‐miR‐21‐5p agomir group (UUO + Mel + agomir);

6: UUO 7 day plus mmu‐miR‐21‐5p agomir group (UUO + agomir).

Mice in group 3, 4 and 5 were intraperitoneal injected with melatonin 20 mg/kg weight at the onset of UUO and 50 mg/kg weight after 6 hours once daily for 7 days.^18^ Meanwhile, mice in group 4, 5 and 6 received tail vein injection of control agomir or mmu‐miR‐21‐5p agomir 3 OD per mouse per week (one injection in total).

After 6 day administration, 24 hours urine sample in six groups was collected using metabolic cages to examine 24 hours urinary protein. Then, UUO mice and sham‐operated control mice were killed. Part of kidney tissue was fixed with 4% paraformaldehyde in 0.1 mol/L cacodylate buffer (pH 7.4), and the remainder was frozen in liquid nitrogen for protein extraction. At the same time, blood samples were collected for evaluation of various renal function indicators, including blood urea nitrogen (BUN) and serum creatinine (SCr).

### 2.7 Histopathological evaluation

Paraffin‐embedded kidney specimens were cut into 4‐µm sections and stained with haematoxylin and eosin (HE) and Masson's trichrome staining. Slides were examined and pictures taken using a Leica DM4B microscope equipped with Leica X software. The degree of collagen accumulation and renal interstitial impairment was assessed as described previously.^19^

### 2.8 Real‐time reverse transcription‐polymerase chain reaction (RT‐PCR)

Total RNA was extracted from cultured NRK‐49F cells and renal tissues using TRIzol reagent (Invitrogen, Cat No. 15596026) to evaluate the mRNA expression. Then, approximately 2.0 µg of total RNA was reversed transcribed into cDNA for RT‐PCR analysis using oligo primers and the Transcriptor First Strand cDNA Synthesis Kit (Roche), according to the manufacturer's instructions. Newly synthesized cDNA was amplified using specific primers and the Fast SYBR Green PCR Master Mix (Applied Biosystems). The sequences of the primers were as follows: α‐SMA: sense, 5′‐GGCATCCACGAAACCACCT‐3′; antisense: 5′‐CCGCCGATCCAGACAGAAT‐3′, miR‐21‐5p: sense, 5′‐GACAAGCTTGCGGCCGCCCTTTAGGAGCATTATGAGCAT‐3′; antisense, 5′‐ATCCTCTAGAGTCGACGAAGGTCAAGTAACAGTCATAC‐3′, β‐actin: sense, 5′‐CACCCGCGAGTACAACCTTC‐3′; antisense: 5′‐CCCATACCCACCATCACACC‐3′, and U6: sense, 5′‐CTCGCTTCGGCAGCACACATATAC‐3′; antisense, 5′‐ACGCTTCACGAATTTGCGTGTC‐3′. The primers were designed and synthesized by Sangon Biotech. β‐actin was used as an endogenous reference control gene for mRNA and U6 RNA for miRNA. The comparative threshold cycle (CT) method was used to evaluate gene relative expression.

### 2.9 Western blot

Western blot analysis was performed according to the previous published paper.^20^ Equal amounts (50 µg) of protein extracts were resolved using 10% sodium dodecyl sulphate‐polyacrylamide gel electrophoresis (SDS‐PAGE). During post‐resolution of the proteins, they were transferred into polyvinylidene difluoride (PVDF) membranes. Membranes were incubated overnight at 4°C with the following primary antibodies: rabbit anti‐α‐SMA (Cat. No ab5694; Abcam; 1:500), mouse anti‐col1a1 (Cat. No sc‐59772; 1:500), mouse anti‐fibronectin (Cat. No sc‐8422; 1:500), rabbit anti‐STAT3 (Cat. No ab31370; Abcam; 1:500), rabbit anti‐STAT3 (phospho Y705) (Cat. No ab76315; Abcam; 1:2000), rabbit anti‐PTEN (Cat. No 51‐2400; Thermo Fisher Scientific, the least detectable dose: 1 µg/mL), rabbit anti‐Spry1 (Cat. No12993; Cell Signaling Technology; 1:1000), rabbit anti‐Smurf2 (Cat. No ab94483; Abcam; 1:500), rabbit anti‐PDCD4 (Cat. No ab51495; Abcam; 1:5000) and rabbit anti‐GAPDH (Cat. No ab9485; Abcam; 1:1000). Post‐incubation with HRP‐conjugated anti‐rabbit IgG or antimouse IgG, the immune‐labelled proteins were detected through enhanced chemiluminescence (ECL; Merck Millipore), and the signal was captured on Biomax L film (Kodak). The intensity of the indicated bands was estimated using ImageJ software.

### 2.10 Statistical analysis

The experimental results were expressed as means ± standard derivation (SD) of at least three independent experiments. Statistical analysis was performed using a Statistical Package for Social Sciences (version 19.0 SPSS Inc). Data were analysed with a two‐sided Student's *t* test, and the one‐way ANOVA followed by a post hoc test. Differences in *P*‐values < .05 were considered statistically significant.

## 3 RESULTS

### 3.1 Effect of melatonin on cell viability in NRK‐49F cells

**Figure 1:**
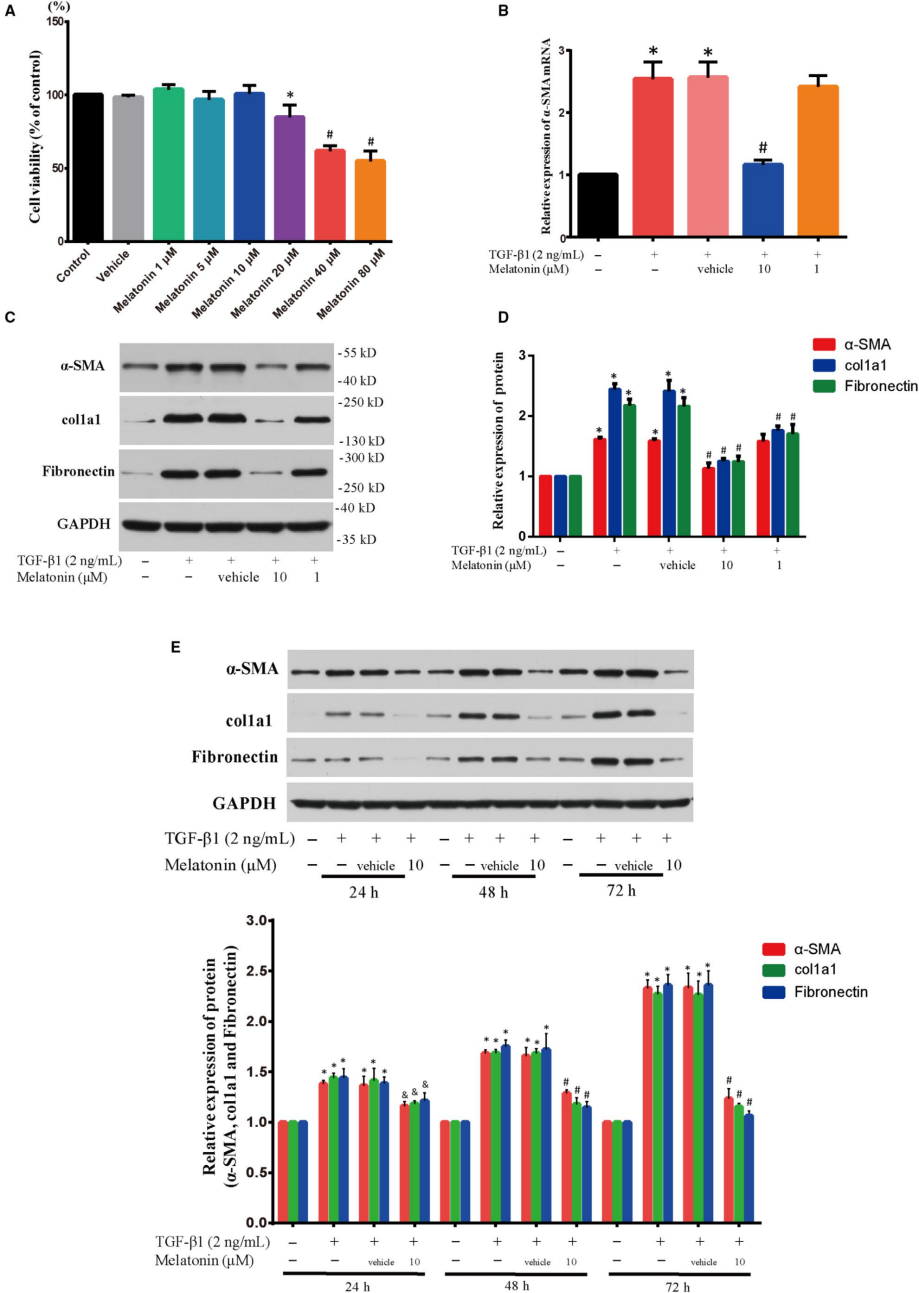
Melatonin alleviated renal fibroblast transdifferentiation and ECM production with TGF‐β1 incubation. A, The effect of melatonin on cell viability was evaluated by CCK‐8 assay (n = 6). NRK‐49F cells were treated with melatonin (1, 5, 10, 20, 40 and 80 µmol/L) for 72 h. The results showed that three different doses of melatonin (1, 5, 10 µmol/L) had no cytotoxicity in the NRK‐49F cells. *P < .05, #*P* < .01 compared with Vehicle group. (B) The level of α‐SMA mRNA in TGF‐β1‐stimulated NRK‐49F cells treated with melatonin (1 and 10 µmol/L) for 48 h was determined by RT‐PCR; n = 6, *P < .01 compared with control group, #*P* < .01 compared with TGF‐β1 + Vehicle group. (C, D) The levels of α‐SMA, col1a1 and fibronectin protein expression in TGF‐β1‐stimulated (2 ng/mL) NRK‐49F cells treated with 1 and 10 µmol/L of melatonin for 48 h were examined by Western blot; n = 6, *P < .01 compared with control group, #*P* < .01 compared with TGF‐β1 + Vehicle group. (E) The levels of α‐SMA, col1a1 and fibronectin protein expression in TGF‐β1‐stimulated NRK‐49F cells treated with 10 µmol/L of melatonin at different time points (24, 48 and 72 h) were assayed by Western blot; n = 6, *P < .01 compared with control group, #*P* < .01, &*P* < .05 compared with TGF‐β1 + Vehicle group

In order to find the proper dosage of melatonin for exploring its role on renal fibroblast transdifferentiation, we used the CCK‐8 assay to analyse the cell survival after treated with various concentrations of melatonin (1, 5, 10, 20, 40, 80 µmol/L) for 72 hours.^21^, ^22^ The results revealed that cell viability in the melatonin treatment groups (1, 5, 10 µmol/L) was not markedly different compared with that of the vehicle group, indicating that these three different doses of melatonin exerted no cytotoxicity in the NRK‐49F cells (Figure 1A). Thus, two dosages (1 and 10 µmol/L) were confirmed for the further experiment of melatonin's effect on renal fibroblast transdifferentiation.

### 3.2 Effect of melatonin on α‐SMA expression in TGF‐β1‐stimulated NRK‐49F cells

Acquiring α‐SMA phenotype is supposed to be a helpful marker for FMT during renal fibrosis.^23^, ^24^ NRK‐49F cells were incubated in the absence or presence of different doses of melatonin (1 or 10 µmol/L) with or without TGF‐β1 (2 ng/mL) treatment for 48 hours; then, the level of α‐SMA mRNA and protein expression was analysed. Our data showed that the level of α‐SMA mRNA and protein was repressed obviously after treatment with 10 µmol/L of melatonin (Figure 1B‐D). However, 1 µmol/L of melatonin did not display any effect. On top of that, NRK‐49F cells were treated with melatonin at a dose of 10 µmol/L at 24, 48 and 72 hours, respectively. As seen in Figure 1E, melatonin reduced the level of α‐SMA protein expression in a time‐dependent manner. Therefore, all these data suggested that melatonin was capable of suppressing renal FMT.

### 3.3 Effect of melatonin on the production of extracellular matrix (ECM) components in TGF‐β1‐stimulated NRK‐49F cells

A notably up‐regulated production of fibronectin and col1a1 (type I collagen) is one important feature of fibroblast differentiation. As the main ingredients of ECM, the expression of col1a1 and fibronectin was evaluated in the present study. Our findings showed that melatonin blocked the improved expression of col1a1 and fibronectin in the TGF‐β1‐treated NRK‐49F cells in a dose‐dependent manner (Figure 1C and D). Furthermore, 10 µmol/L of melatonin reduced the level expression of both proteins with time (Figure 1E).

Although 1 µmol/L of melatonin (low dose) had no effect on the expression of α‐SMA mRNA and protein in the TGF‐β1‐treated NRK‐49F cells, it inhibited the production of ECM components (Figure 1C and D). The results suggested even low dose of melatonin can exert an inhibitory effect on fibroblast activation. In a word, our findings indicated that melatonin could attenuate the differentiation of renal fibroblast and prevent ECM production after TGF‐β1 treatment.

### 3.4 Effect of melatonin on α‐SMA filament formation in NRK‐49F cells treated with TGF‐β1

**Figure 2:**
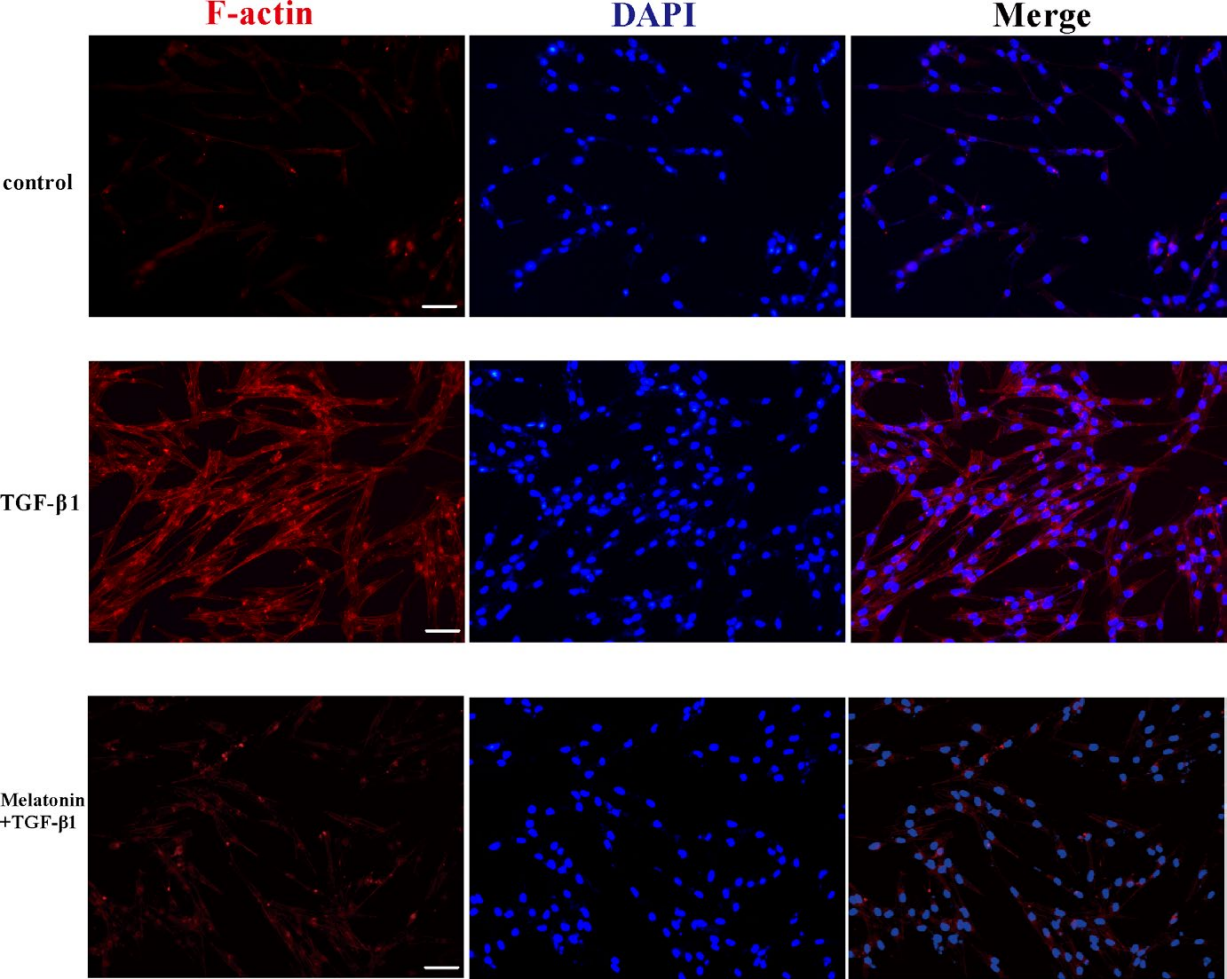
Melatonin decreased the formation of stress fibre in TGF‐β1‐stimulated NRK‐49F cells. The formation of stress fibre in TGF‐β1‐stimulated NRK‐49F cells treated with or without 10 µmol/L of melatonin for 48 h was evaluated by cell immunofluorescence staining. Magnification ×400, Scale bar = 50 µm

FMT is characterized by the occurrence of incrassate α‐SMA‐inclusive cytoplasmic filaments.^25^ The results from filament staining displayed that melatonin (10 µmol/L dose) obviously repressed stress fibres formation and F‐actin level (Figure 2 and Figure S1). These observations indicated that melatonin could arrest a transition from fibroblast phenotype to myofibroblast phenotype.

### 3.5 Effect of melatonin on the miR‐21‐5p expression in NRK‐49F cells treated with TGF‐β1

**Figure 3:**
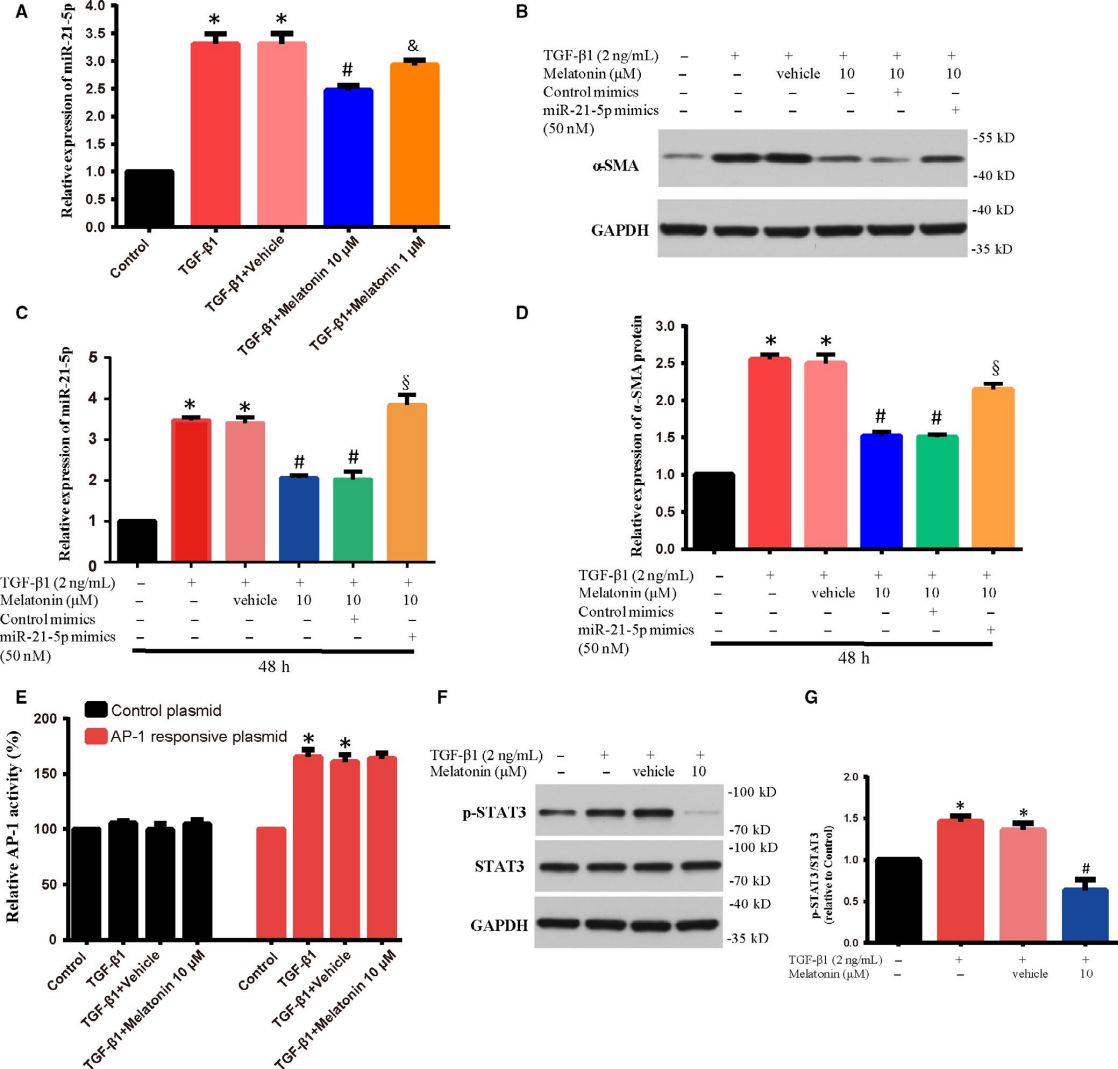
The level of α‐SMA protein and miR‐21‐5p was examined in NRK‐49F cells. A, The level of miR‐21‐5p expression in TGF‐β1‐stimulated (2 ng/mL) NRK‐49F cells treated with 1 and 10 µmol/L of melatonin for 48 h was examined by RT‐PCR; n = 6, *P < .01 compared with control group, #*P* < .01, &*P* < .05 compared with TGF‐β1 + Vehicle group. (B‐D) The level of α‐SMA protein and miR‐21‐5p was examined in NRK‐49F cells transfected with miR‐21‐5p mimics (50 nmol/L) or control mimics, and then co‐cultured with TGF‐β1 (2 ng/mL) with or without 10 µmol/L of melatonin for 48 h; n = 6, *P < .01 compared with control group, #*P* < .01 compared with TGF‐β1 + Vehicle group, §*P* < .01 compared with TGF‐β1 + melatonin + control mimics. (E) The level of AP‐1 responsive plasmid luciferase activity was measured in NRK‐49F cells co‐treated with TGF‐β1 (2 ng/mL) and 10 µmol/L of melatonin for 48 h. *P < .01 compared with control group. (F, G) The level of p‐STAT3 protein was determined by Western blot in NRK‐49F cells co‐treated with TGF‐β1 (2 ng/mL) and 10 µmol/L of melatonin for 48 h; n = 6, *P < .01 compared with control group, #*P* < .01 compared with TGF‐β1 + Vehicle group

There is some evidence that elevation of renal miR‐21 plays an important role in regulating expression of ECM and fibroblast transdifferentiation during renal fibrosis.^26^ However, whether the effect of melatonin against fibrosis was associated with miR‐21‐5p has not been reported. To determine the role of miR‐21‐5pin melatonin‐blocked renal FMT, we evaluated the miR‐21‐5p expression in NRK‐49F cells co‐treated with TGF‐β1 and melatonin (1 or 10 µmol/L dose) for 48 hours. As shown in Figure 3A, miR‐21‐5p expression significantly decreased in TGF‐β1‐stimulated NRK‐49F cells after melatonin administration. These findings indicated that melatonin could inhibit the miR‐21‐5p expression, which might be associated with the inhibitory effect of melatonin on renal FMT.

### 3.6 The inhibitory effect of melatonin on α‐SMA expression induced by TGF‐β1 was reversed by miR‐21‐5p mimics

In order to verify melatonin works by inhibiting the miR‐21‐5p signal, we transfected a miR‐21‐5p mimics into NRK‐49F cells. Then, NRK‐49F cells were co‐treated with TGF‐β1 and melatonin (10 µmol/L dose) for another 48 hours. The results found that the level of α‐SMA protein was remarkably up‐regulated in TGF‐β1‐treated NRK‐49F cells co‐incubation with melatonin and miR‐21‐5p mimics compared to those of co‐incubation with melatonin and control mimics (Figure 3B‐D). Our data revealed that up‐regulation of miR‐21‐5p expression could partially block the inhibitory effect of melatonin on α‐SMA, indicating that melatonin could alleviate renal FMT through down‐regulating miR‐21‐5p expression.

### 3.7 Effect of melatonin on TGF‐β1‐induced STAT3 activation and AP‐1 activity

It has been demonstrated that several transcription factors can significantly up‐regulate miR‐21‐5p expression, for example activation protein 1 (AP‐1 family) and STAT3.^27^ To investigate whether melatonin‐mediated change of transcription factors activities participated in the biological consequences of melatonin in TGF‐β1‐induced renal fibroblast differentiation, we detected STAT3 phosphorylation level and AP‐1 activity. As shown in Figure 3E, AP‐1 activity was markedly increased after TGF‐β1 stimulation, which was not changed after melatonin treatment. Next, the detection results of STAT3 phosphorylation showed that melatonin administration apparently inhibited the activation of STAT3 (Figure 3F and G). Altogether, our findings indicated that melatonin might down‐regulate miR‐21‐5p expression through blocking STAT3 activation induced by TGF‐β1, but not AP‐1 activity in NRK‐49F cells. Moreover, in order to determine whether STAT3 activation was important for miR‐21‐5p expression, we evaluated miR‐21‐5p expression in NRK‐49F cells relative to STAT3 activation status. Activation by tyrosine phosphorylation at residue 705 (Y705) is required for STAT3 to induce downstream changes in gene expression in response to cytokine stimulation.^28^ We therefore confirmed the effect of a specific small molecular inhibitor of STAT3 phosphorylation: Stattic.^29^ The results showed that Stattic did not affect the basal expression level of miR‐21‐5p in untreated NKK‐49F cells (Figure S2), suggesting that Stattic itself was unable to modulate miR‐21‐5p expression.

### 3.8 Effect of melatonin on miR‐21‐5p targets expression

**Figure 4:**
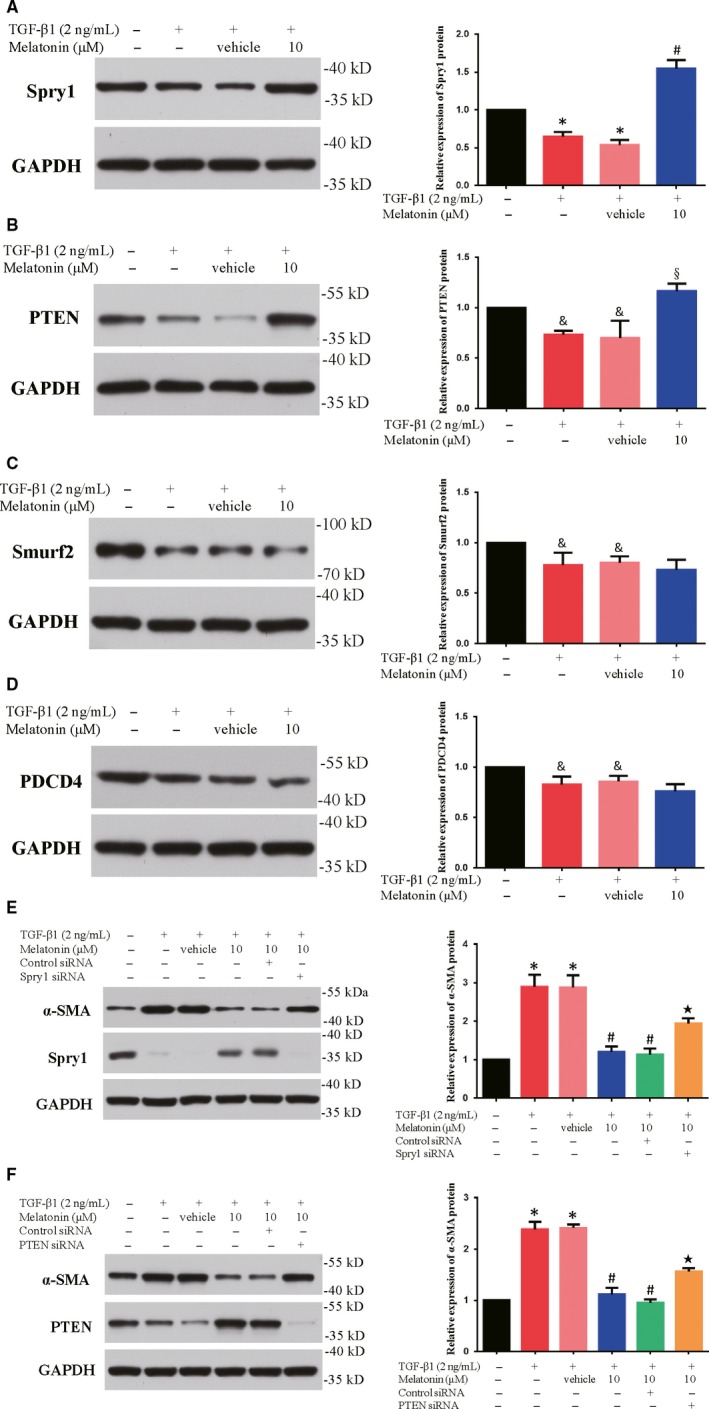
Melatonin increased the miR‐21‐5p targets Spry1 and PTEN expression, but not Smurf2 or PDCD4. The levels of Spry1 (A), PTEN (B), Smurf2 (C) and PDCD4 (D) protein expression were examined by Western blot inNRK‐49F cells co‐treated with TGF‐β1 (2 ng/mL) and 10 µmol/L of melatonin for 48 h; n = 6, *P < .01, &*P* < .05 compared with control group, #*P* < .01, §*P* < .05, compared with TGF‐β1 + Vehicle group. The level of α‐SMA protein in NRK‐49F cells was evaluated by Western blot. NRK‐49F cells were transfected with Spry1 siRNA/control siRNA (E) and PTEN siRNA/control siRNA (F), respectively, and then co‐cultured with TGF‐β1 (2 ng/mL) with or without 10 µmol/L of melatonin for 48 h; n = 6, *P < .01 compared with control group, #*P* < .01 compared with TGF‐β1 + Vehicle group, ★*P* < .01 compared with TGF‐β1 + melatonin + control siRNA

Depending on the availability of a certain number of mRNA targets, a single miRNA may regulate multiple pathways and lead to multiple phenotypes in different cellular environments.^30^ Target validation experiments demonstrated that phosphatase and tensin homolog (PTEN) and Sprouty1 (Spry1) are two of potential targets of miR‐21.^31^, ^32^ Sprouty1 (Spry1), a negative regulator of ERK/MAPK signalling, has been revealed to be associated with increased fibrogenic epithelial‐to‐mesenchymal transition of TGF‐β1.^33^ PTEN negatively regulates Akt/PKB activation, which has been implicated in renal fibrosis.^34^ To explore whether melatonin treatment works through modulating miR‐21‐5p targets, firstly we examined the protein expression of Spry1 and PTEN. The results showed the level of Spry1 and PTEN protein was evidently improved after melatonin administration (Figure 4A and B). Additionally, another two miR‐21‐5p targets, Smurf2 and PDCD4, were important factors for fibroblast differentiation and fibrogenic process.^35^, ^36^ However, after detecting the protein level of Smurf2 and PDCD4, we found melatonin did not significantly affect their expression (Figure 4C and D). These data indicated that melatonin treatment could regulate miR‐21‐5p targets Spry1 and PTEN expression, which might lead to the inhibition of fibroblast differentiation.

### 3.9 The inhibitory effect of melatonin on α‐SMA expression induced by TGF‐β1 required Spry1 and PTEN

From the above‐mentioned data, we supposed the increased level of Spry1 and PTEN protein expression might be related to the inhibitory effects of melatonin. As a consequence, we evaluated the influence of Spry1 and PTEN in the inhibitory effect of melatonin on α‐SMA expression in TGF‐β1‐treated NRK‐49F cells. Firstly, we verified the transfection efficiency of Spry1 siRNA and PTEN siRNA in NRK‐49F cells. The results showed that Spry1 and PTEN protein expression was significantly inhibited after Spry1 siRNA and PTEN siRNA transfection (Figure S3). Seen from Figure 4E and F, administration of Spry1 siRNA in NRK‐49F cells reversed the consequences of melatonin on expression of α‐SMA. Similarly, PTEN siRNA treatment partially restored the α‐SMA expression which was blocked by melatonin. These findings suggested that the elevated Spry1 and PTEN might be required for melatonin function on renal fibroblast differentiation.

### 3.10 The renoprotective effect of melatonin was reversed by miR‐21‐5p agomir in UUO mice

Compared with sham group, the mice after 7 days of UUO showed significantly elevated BUN and SCr values, as well as 24 hours urinary protein levels (Table 1), suggesting that UUO caused renal dysfunction. Unsurprisingly, melatonin treatment markedly reduced the up‐regulation of BUN, SCr and 24‐hour urinary protein caused by UUO. However, the protective effect of melatonin on renal function was reversed after the additional miR‐21‐5p agomir injection in group 5. Despite this, compared with the UUO + agomir group (group 6), melatonin could still promote the recovery of renal function to a certain extent in agomir‐injected UUO mice (group 5). These data suggested that miR‐21‐5p might be involved in the pathophysiological process of renoprotection by melatonin.

**Table 1:** Parameters of renal function in each group

Group	N	Serum creatinine (mmol/L)	Blood urea nitrogen (µmol/L)	Urinary protein (mg/24 h)
Sham	8	33.4 ± 10.34	6.43 ± 0.75	20.34 ± 4.92
UUO	8	72.7 ± 8.22^a^	18.68 ± 2.09^a^	83.09 ± 15.13^a^
UUO + Mel	8	51.2 ± 3.65^b^	10.15 ± 1.17^b^	45.28 ± 6.06^b^
UUO + Mel + NC	8	51.9 ± 3.78^c^	9.76 ± 1.64^c^	45.6 ± 5.99^c^
UUO + Mel + agomir	8	64.6 ± 4.58^d^	18.34 ± 1.80^d^	82.44 ± 13.91^d^
UUO + agomir	8	93.2 ± 6.22^b^, ^c^	25.7 ± 1.19^b^, ^c^	102.3 ± 10.1^b^, ^c^

Note

Each value is expressed as mean ± standard deviation (SD). Values sharing following symbols differ significantly.

Abbreviations: Mel, melatonin; NC, control agomir; UUO, unilateral ureteral obstruction.

^a^ *P* < .01 vs the Sham group.

^b^ *P* < .01 vs the UUO group.

^c^ *P* < .01 vs the UUO + Mel + agomir group.

^d^ *P* < .01 vs the UUO + Mel group.

**Figure 5:**
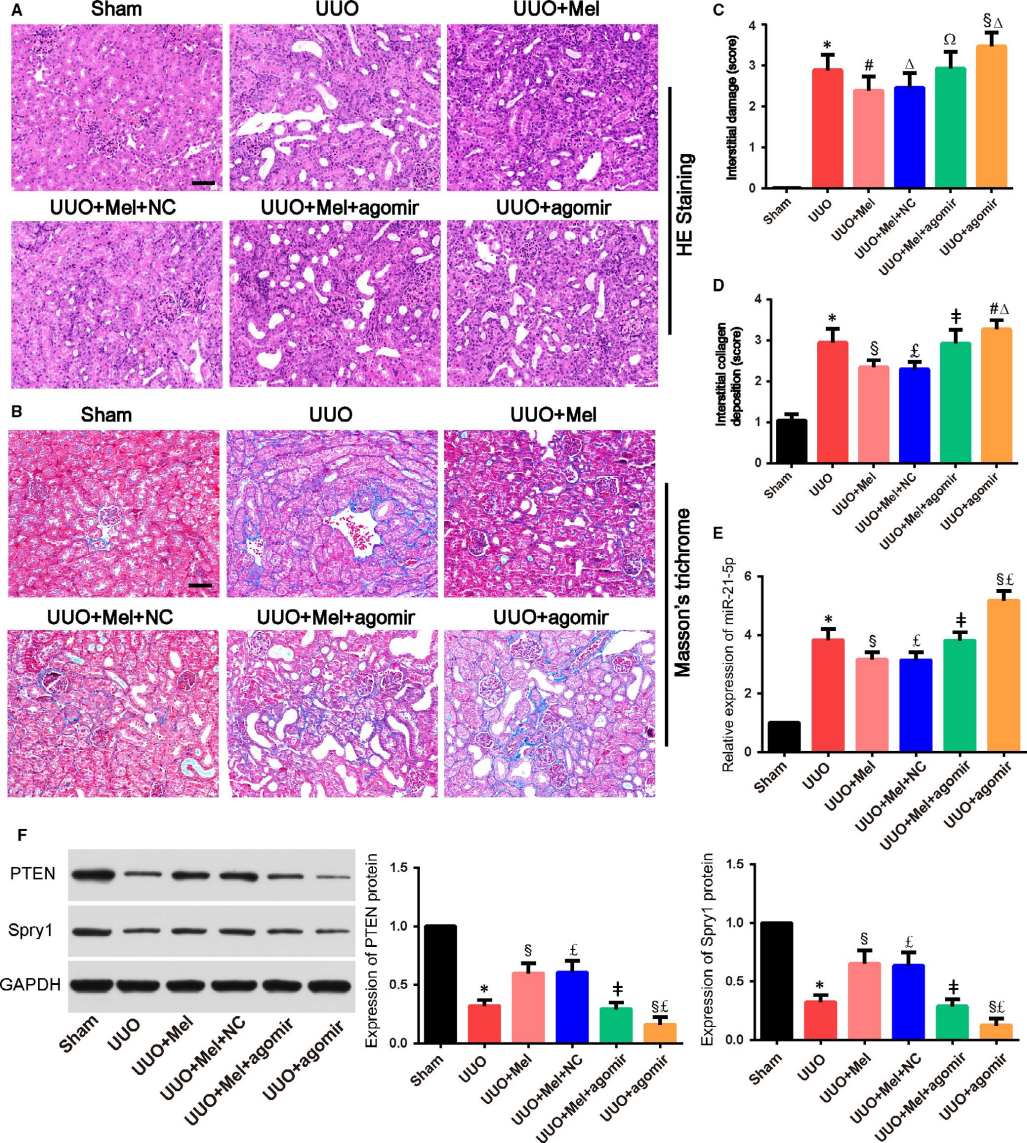
MiR‐21‐5p alleviated renoprotective effects of melatonin and inhibited up‐regulation of Spry1 and PTEN induced by melatonin in the kidney tissues of unilateral ureteral obstruction (UUO) mice. A, Renal tubular injury was assessed by haematoxylin and eosin (HE) staining (×200). Scale bar = 50 µm. B, The deposition of collagen in kidney tissues of UUO rats was determined by Masson's trichrome staining (×200). Blue (aniline blue) represents collagen fibres; red (acid fuchsin) represents muscle fibres. Scale bar = 50 µm. C and D, The statistical analyses of tubulointerstitial damage scores and the degrees of interstitial collagen deposition in different groups. E, RT‐PCR analysis of the expression of miR‐21‐5p in kidneys of mice in different group. F, Western blot analysis of the expression of PTEN and Spry1 in different groups. Bars represent means ± standard deviation (SD) (n = 8). *P < .01 vs the Sham group; #*P* < .05, §*P* < .01 vs the UUO group; Δ*P* < 0.05, £*P* < .01 vs the UUO + Mel + agomir group; Ω*P* < 0.05, ǂ*P* < 0.01 vs the UUO + Mel group. Mel, melatonin; NC, control agomir

We further investigated the protective effect of melatonin against obstructive nephropathy. Normal kidney tissue structure is necessary to maintain normal kidney function. Histological examination showed that there were obvious tubular dilatation and atrophy, renal interstitial widening, interstitial inflammatory cells infiltration and a large amount of collagen deposition in the kidneys of UUO mice (Figure 5A and B). However, in the melatonin alone treated group, these above histopathological changes, including interstitial fibrosis and collagen deposition, were markedly improved compared with those in the UUO group (Figure 5C and D). In addition, consistent with the results of in vitro studies, the expression of miR‐21‐5p in the kidneys of UUO mice increased significantly, and the expression of its target genes PTEN and Spry1 was significantly down‐regulated, which were significantly improved after melatonin administration (Figure 5E and F). MiR‐21‐5p agomir could block the above‐mentioned effects of melatonin and manifested as up‐regulation of miR‐21‐5p and down‐regulation of PTEN and Spry1 (Figure 5E and F). It is worth to mention that compared with the UUO + agomir group, the expression of miR‐21‐5p in the UUO + Mel + agomir group was significantly reduced, which suggested that melatonin could regulate the miR‐21‐5p expression in the kidneys of UUO mice (Figure 5E). Our results implied that regulation of miR‐21‐5p might participate in the anti‐fibrotic effect of melatonin in UUO mice.

**Figure 6:**
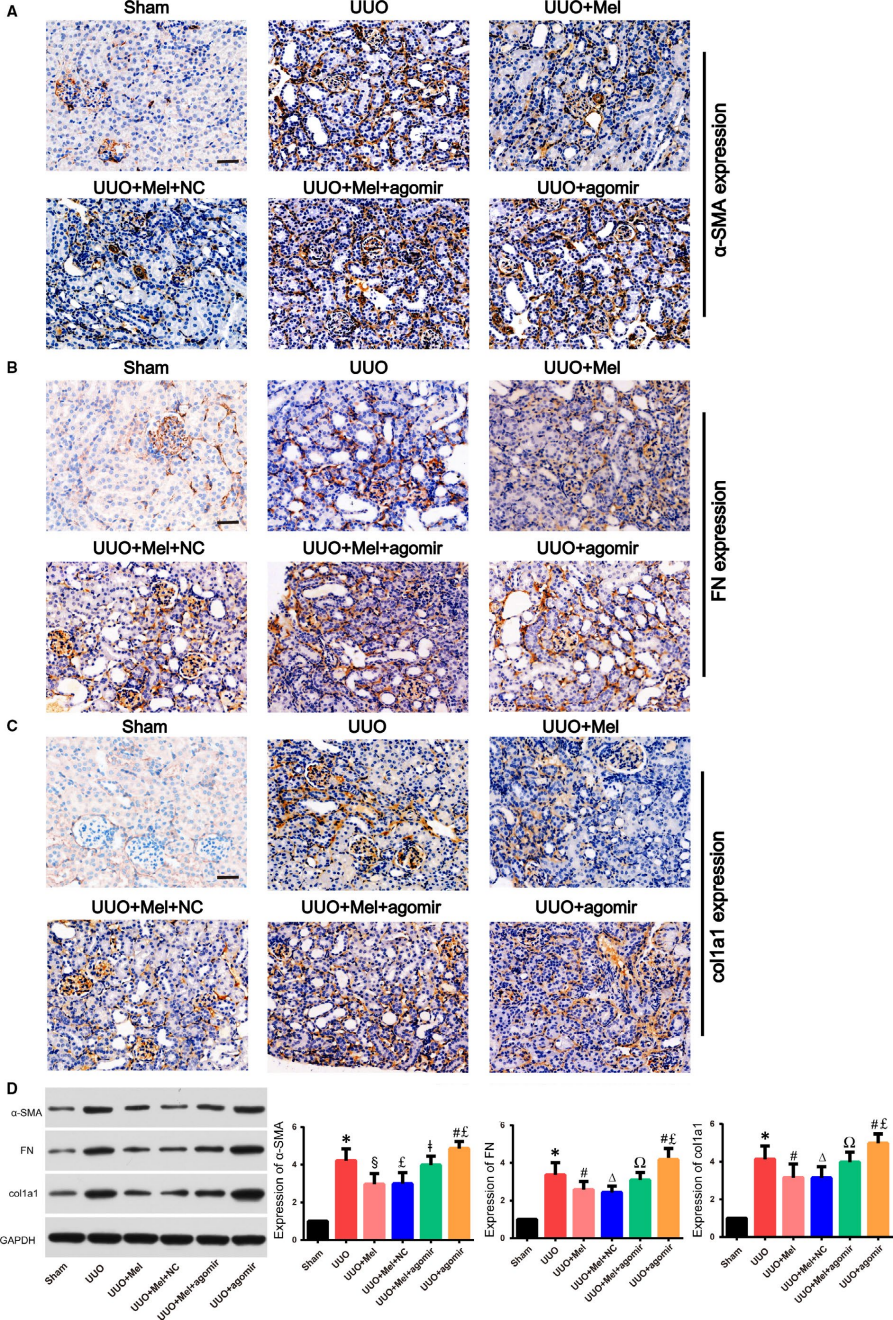
MiR‐21‐5p inhibited down‐regulation of α‐SMA, FN and col1a1 induced by melatonin in unilateral ureteral obstruction (UUO) mice. The expression of α‐SMA (A), FN (B) and col1a1 (C) in different groups was determined by immunohistochemical staining (×200). Scale bar = 50 µm. (D) Western blot showed that protein expression levels of α‐SMA, FN and col1a1in different groups. Bars represent means ± standard deviation (SD) (n = 8). *P < .01 vs the Sham group; #*P* < .05, §*P* < .01 vs the UUO group; Δ*P* < 0.05, £*P* < .01 vs the UUO + Mel + agomir group; Ω*P* < 0.05, ǂ*P* < 0.01 vs the UUO + Mel group. Abbreviation: Mel, melatonin; NC, control agomir; FN, fibronectin

Furthermore, the expression of α‐SMA, fibronectin and col1a1 increased prominently in kidneys of UUO model (Figure 6). Melatonin treatment showed a good inhibitory effect on UUO‐induced up‐regulation of α‐SMA, fibronectin and col1a1 (Figure 6). However, miR‐21‐5p agomir obviously abrogated the anti‐fibrotic action of melatonin compared with the UUO + Mel group. In addition, compared with the UUO + agomir group, melatonin in the UUO + Mel + agomir group still showed a certain degree of anti‐fibrotic ability, including reducing fibrotic regions and down‐regulating the expression of fibrosis‐related proteins.

## 4 DISCUSSION

In the current study, we investigated the inhibitory action of melatonin on renal FMT in TGF‐β1‐exposed NRK‐49F cells and the renoprotective effect of melatonin on renal fibrosis in UUO mice. According to the analysis of the current experimental results, we firstly revealed that melatonin significantly prevented the expression of miR‐21‐5p in NRK‐49F cells incubated with TGF‐β1 and kidneys of UUO mice. Conversely, miR‐21‐5p mimics and agomir abrogated the anti‐fibrotic effects of melatonin in vitro and in vivo. In addition, we found that melatonin significantly down‐regulated STAT3 phosphorylation and up‐regulated the expression of miR‐21‐5p target genes PTEN and Spry1 in NRK‐49F cells incubated with TGF‐β1 and kidneys of UUO mice.

As we all known, FMT is a key event for aberrant tissue repair, leading to the pathological alterations that determine renal fibrosis. Targeting renal myofibroblasts has been recognized as promising potential strategy for renal fibrosis. It has been demonstrated that atrasentan and sparsentan (anti‐fibrotic drugs) successfully delay the progress of renal fibrosis through inhibiting activation of fibroblasts and transdifferentiation.^37^ Therefore, blocking fibroblast transdifferentiation may effectively reduce the progression of renal failure, providing more treatment options for CKD patients. For the first time, our data showed that 10 µmol/L melatonin (a safe dose in rat renal fibroblast NRK‐49F cells) treatments can partially prevent α‐SMA mRNA and protein expression, as well as formation of α‐SMA microfilaments induced by TGF‐β1 in NRK‐49F cells. These results indicated that melatonin may have an inhibitory effect on FMT in renal fibroblasts response to TGF‐β1 induction.

In fibrogenesis, fibroblasts are thought to transform to the so‐called myofibroblasts, during which accompanied with the accumulation of numerous ECM components including fibronectin and col1a1.^10^ The excessive ECM production in the interstitial area is the important pathological character of renal fibrosis and results in progressive renal function decline.^38^ Thus, targeting ECM production might be an effective management to slow the fibrotic progress. The current study revealed that melatonin can decrease the contents of col1a1 and fibronectin in dose‐ and time‐dependent manners in renal fibroblasts response to TGF‐β1 stimulation. In vivo studies further verified the in vitro findings that melatonin treatment could markedly improve renal interstitial fibrosis and collagen deposition caused by UUO, and reduced the expression of α‐SMA, col1a1 and fibronectin in kidneys of UUO mice. Interestingly, 1 µmol/L of melatonin (low dose) had no effect on the expression of α‐SMA mRNA and protein in the TGF‐β1‐treated NRK‐49F cells, but it inhibited the production of ECM components. The results suggested even low dose of melatonin can exert an inhibitory effect on fibroblast activation.

MicroRNAs (miRNAs) are endogenous short‐stranded non‐coding RNAs, which can down‐regulate the mRNA expression of their target genes through post‐transcriptional inhibition, thereby affecting and participating in important cellular biological functions. In the kidney, miRNAs are shown to be involved in regulating the physiological functions of the kidney. The results of clinical and animal experiments indicate that miRNAs play vital roles in the pathophysiology of kidney‐related diseases.^26^ It has been confirmed in many experimental models of renal fibrosis that the increased expression of miR‐21 is closely related to the progression of fibrosis.^11^, ^14^, ^34^ These results indicated that miR‐21 may serve as a potential candidate against renal fibrosis. The findings from the present study implicated that melatonin could effectively prevent miR‐21‐5p expression in renal fibroblasts response to TGF‐β1 incubation and kidneys of UUO mice. Administration of miR‐21‐5p mimics/agomir was found to abolish the inhibitory action of melatonin on FMT and reverse the anti‐fibrotic effect of melatonin on UUO mice. Herein, we inferred that the inhibitory effects of melatonin on FMT and fibrosis were related, at least in part, to its regulation of miR‐21‐5p expression in renal fibroblasts. Furthermore, it has been demonstrated that transcription factors AP‐1 and STAT3 can significantly promote miR‐21‐5p expression,^39^, ^40^ which is closely associated with renal fibrogenesis.^41^, ^42^ Our current study found that melatonin could suppress transcription factor STAT3 activity, but not AP‐1 activity. In addition, we found that Stattic (an inhibitor of STAT3 phosphorylation) did not affect the basal expression level of miR‐21‐5p in untreated NKK‐49F cells. These data suggested that melatonin might mediate miR‐21‐5p expression through down‐regulating STAT3 activity during fibroblast transdifferentiation. STAT proteins belong to a superfamily controlling transcription‐regulating signalling and the response of cells to environmental stimuli in the absence of protein synthesis through the STAT pathway.^43^ In mammals, STAT3 has been widely investigated due to its numerous functions, including cell growth regulation and inflammation.^44^ STAT3 is activated by tyrosine phosphorylation, predominantly JAK, leading to its phosphorylation on activating tyrosine residues, dimerization and nuclear translocation.^43^ The pharmacological effects of melatonin have been reported to be closely related to JAK2/STAT3 signalling.^22^, ^45^ Yang et al showed that melatonin exerted protective effects on heart by reducing ischaemia/reperfusion‐induced mitochondrial oxidative damage via the activation of JAK2/STAT3 signalling.^45^ JAK2/STAT3 signalling pathway activation by melatonin during diabetic nephropathy was also demonstrated by Ji and colleagues that confirmed decrease in apoptotic podocyte number, down‐regulation in Bax level, reduction in oxidative stress damage and alleviation in inflammation after melatonin treatment.^22^ In the present study, we observed that melatonin can significantly inhibit the activation of STAT3 phosphorylation in the TGF‐β1‐treated NRK‐49F cells, but its underlying mechanism still needs further investigation.

Moreover, target validation experiments have revealed that PTEN and Spry1 are target genes of miR‐21‐5p.^31^, ^32^ MiR‐21‐5p has been found to inhibit PTEN to increase PI3K and Akt phosphorylation, and then promotes MMP‐2 overexpression.^32^ As Spry1 is an inhibitor of Ras/MEK/ERK, inhibition of Spry1 by miR‐21‐5p will activate ERK to promote TGF‐β1 induced fibrosis.^46^ Our findings showed that melatonin treatment significantly up‐regulated the level of PTEN and Spry1 protein expression in renal fibroblasts response to TGF‐β1 induction and kidneys of UUO mice, indicating that PTEN and Spry1 protein may participate in the effects of melatonin on renal fibroblast transdifferentiation and renal fibrosis. As a consequence, our present exploration revealed that melatonin could ameliorate renal FMT and renal fibrosis via miR‐21‐5p/PTEN and/or miR‐21‐5p/Spry1 signalling pathway, which may be a vital anti‐fibrotic mechanism of melatonin.

In conclusion, the present study confirmed that melatonin could alleviate renal FMT and renal fibrosis. Further analysis of the underlying mechanisms implied that melatonin protected renal against fibrosis through reducing expression of miR‐21‐5p, which might be related to decreased phosphorylation of STAT3. Regulation of miR‐21‐5p/PTEN and/or miR‐21‐5p/Spry1 signalling pathway is also participated in the anti‐fibrotic action of melatonin in kidney. These signals could restore PTEN and/or Spry1 expression and prevent the expression of pro‐fibrotic genes. The present study puts forward a novel mechanism for melatonin action and further provides new evidence on the anti‐fibrotic effects of melatonin. However, more detailed and in‐depth studies on melatonin, miR‐21‐5p and signal pathways need further exploration in the future.

## CONFLICT OF INTEREST

The authors confirm that there are no conflicts of interest.

## AUTHOR CONTRIBUTIONS

ZL conceived and designed the study. NL FG and YL performed the experiments. NL FG and JZ analysed the specimens. NL and ZL wrote and prepared the original draft.

## Supporting information

Fig S1

Fig S2

Fig S3

## Data Availability

The data that support the findings of this study are available from the corresponding author upon reasonable request.
