# Supplementary figures and images for: Melatonin ameliorates renal fibroblast‐myofibroblast transdifferentiation and renal fibrosis through miR‐21‐5p regulation
Source: J Cell Mol Med. 2020 Apr 3;24(10):5615–28. doi: 10.1111/jcmm.15221 (PMC7214152; doi:10.1111/jcmm.15221)

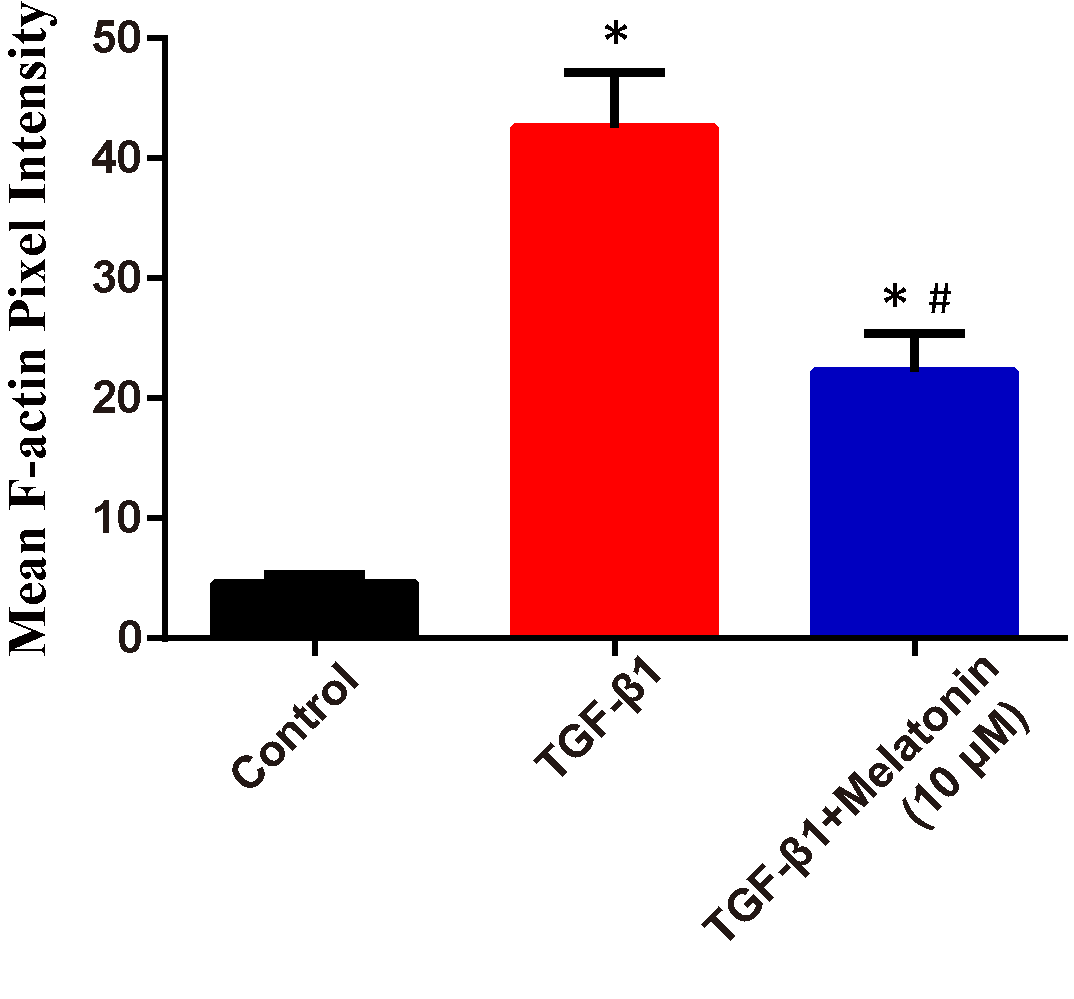

Supplement: Supplementary file 1 — Fig S1 [file JCMM-24-5615-s001.tif]

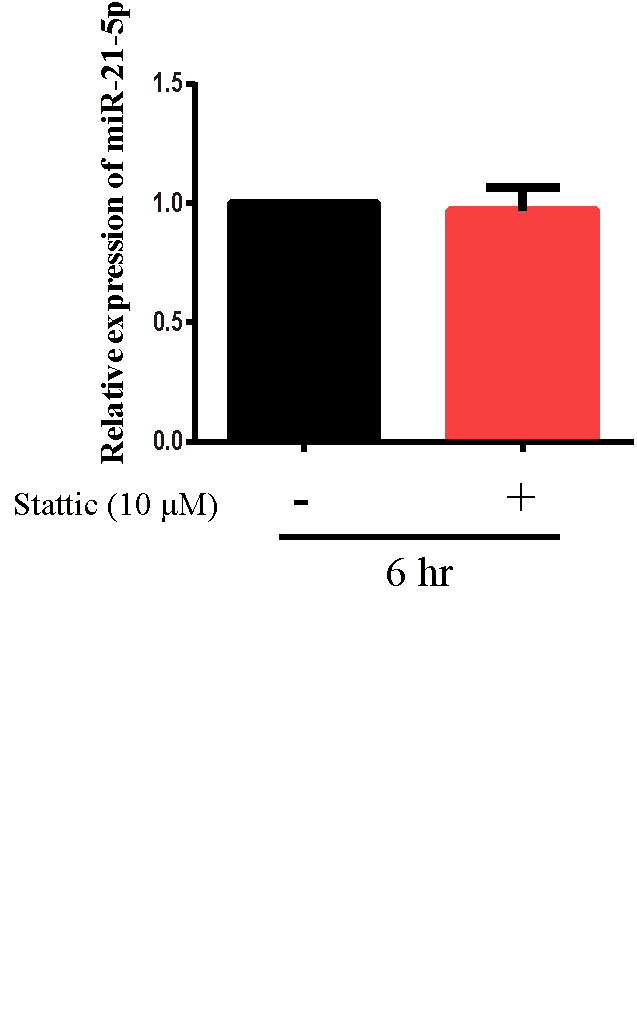

Supplement: Supplementary file 2 — Fig S2 [file JCMM-24-5615-s002.tif]

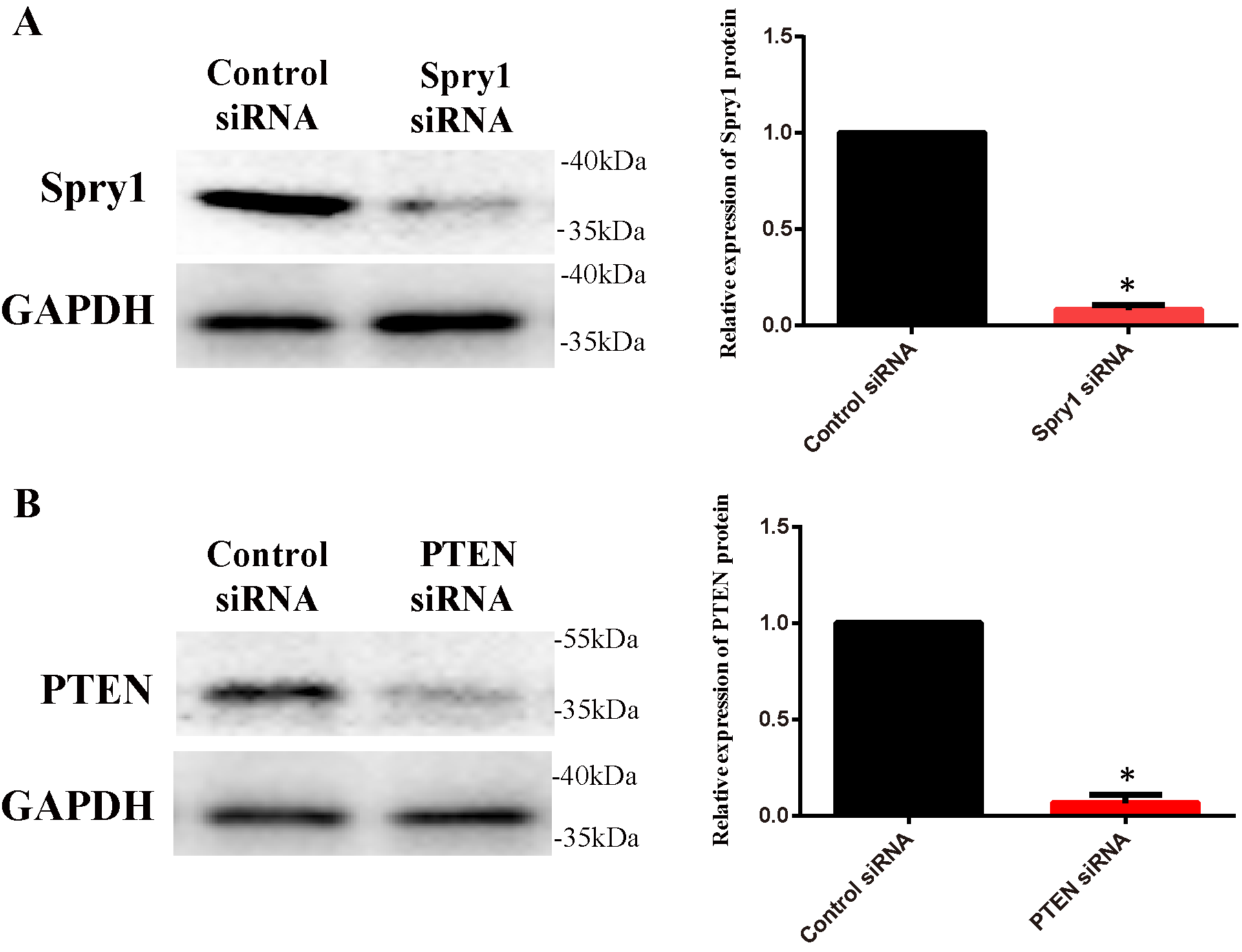

Supplement: Supplementary file 3 — Fig S3 [file JCMM-24-5615-s003.tif]
